# Cardiovascular disease and depression: a narrative review

**DOI:** 10.3389/fcvm.2023.1274595

**Published:** 2023-11-21

**Authors:** Xinzhong Li, Jiahui Zhou, Min Wang, Chengmin Yang, Guibo Sun

**Affiliations:** Institute of Medicinal Plant Development, Peking Union Medical College and Chinese Academy of Medical Sciences, Beijing, China

**Keywords:** depression, cardiovascular disease, pathogenesis, epidemiology, treatment

## Abstract

In clinical practice, it is frequently observed that cardiac and psychological disorders frequently co-occur, leading to the emergence of a field known as cardiovascular disease with depression. Depression, in particular, poses a remarkable risk for the evolution of cardiovascular disease and intimately relates to adverse cardiovascular outcomes and mortality. Moreover, individuals who are depressed exhibit a higher susceptibility to developing cardiovascular disease compared to those in good health. Patients diagnosed with cardiovascular disease with depression disease face a heightened risk of mortality within a 5-year timeframe, and their prognosis remains unsatisfactory even after receiving treatment targeting a single disorder, with a notable recurrence rate. Psychological interventions in conjunction with medications are commonly employed in clinical settings for treating patients with cardiovascular disease and depression diseases, albeit with limited effectiveness and unfavorable prognosis. Traditional Chinese medicine (TCM), such as Shuangxinfang, Chaihujialonggumuli, and Yixin Ningshen Tablet, etc., have been reported and have Therapeutic effects in patients with cardiovascular disease combined with depression. Despite numerous articles documenting a notable association between heart disease and depression, there exists a dearth of studies elucidating the precise pathogenesis and target of action for cardiovascular disease with depression diseases. This article endeavors to consolidate the epidemiological data, potential pathogenic mechanisms, and available treatment modalities for cardiovascular disease with depression diseases. Its primary objective is to unveil plausible co-morbid mechanisms and suitable treatment approaches, thereby offering novel insights for the prevention, diagnosis, and management of cardiovascular disease with depression diseases.

## 1. Introduction

Research has demonstrated that individuals diagnosed with depression exhibit an increased susceptibility to cardiovascular disease. Conversely, patients with cardiovascular disease tend to experience comorbid depression. These two conditions are mutually causative and exert reciprocal effects on one another, thus constituting a significant health concern. This phenomenon is clinically referred to as cardiovascular disease with depression diseases (1). For a very long time, it has been known that mental illness and cardiovascular disease are related. However, it has only recently been acknowledged that depression is a risk factor for coronary heart disease and coronary heart disease increases the prevalence of depression (2). Notably, anxiety and depression serve as risk factors for coronary heart disease development, with respective prevalence rates of 21% and 13% for concurrent occurrence (3). The gradual buildup of atherosclerosis over an extended duration culminates in coronary artery disease (CAD), which clinically presents as either acute coronary syndromes or stable angina. Stable coronary artery disease encompasses a diverse range of coronary pathophysiology, encompassing both obstructive and nonobstructive coronary artery disease, resulting in angina and induced ischemia (4). Depression is a mental illness characterized by low mood and a lack of pleasure, reducing the quality of life for many people. As stated by the World Health Organization, around 5% of people worldwide experience depression. According to some gloomy projections, it will be the main contributor to the heavy load of disease by the year 2030 (5). Numerous popular clinical trials and epidemiological studies have found a definite correlation between coronary heart disease and depression. Also, a secondary analysis of clinical trials has shown that when depression improves, the prognosis for coronary heart disease also improves (2).

Depression is correlated with cardiovascular disease morbidity and mortality (6). The prevalence of depressed patients with coronary artery disease is 20%–40%, much higher than average for healthy people (7). One analysis assesses the morbidity and persistence of melancholia in patients who suffer from acute myocardial infarction (AMI) and shows that mental disorder is common and sustained in AMI survivors (8). For coronary artery disease patients who suffer from anxiety or depression, the clinical treatment is based on conventional treatment such as thrombolysis/intervention and secondary prevention of coronary artery disease, with supplementary psychotherapy (9). Escitalopram has also been reported for the treatment of patients with coronary artery disease combined with depression (10). A relevant randomized controlled trial, encompassing 42 trials on the treatment of depression in patients with heart disease combined, reports that selective serotonin reuptake inhibitors (SSRIs) appear to be secure in patients with cardiovascular illness and profitable for patients with cardiovascular disease with depression disease. Psychotherapy proves to be useful for depression in coronary artery disease and heart failure, although there was less evidence of a therapeutic impact in these conditions (11). Formononetin may be a promising treatment since it targets GSK-3 to control macrophage/microglia polarization, which enhances heart function and reduces depressed behavior in mice following myocardial infarction depression (12). A recent study suggests that Sestrin2 reduces inflammation and iron death through LKB1-mediated AMPK activation in rats of myocardial infarction combined with depression, potentially a potential therapeutic target (13).

In conclusion, the incidence of cardiovascular disease combined with depression is high, the diagnosis rate is low, the prognosis is poor, the therapeutic target is still unclear, and the exceptional pathogenesis has not been fully expounded. This article reviews the epidemiology and potential co-morbid mechanisms of coronary heart disease and depression. Then, it looks forward to the future direction of the development of research on the pathogenesis and treatment of cardiovascular disease with depression disease, as well as providing novel opinions for the prevention, diagnosis, and treatment of cardiovascular disease with depression disease.

## 2. Methods

PubMed was used to obtain the publications, and the retrieval strategy was [TS = (“cardiovascular disease” AND depression)], [TS = (“cardiovascular disease” AND “depression” AND “HPA axis”)], [TS = (“cardiovascular disease” AND “depression” AND “Inflammation”)], [TS = (“cardiovascular disease” AND “depression” AND “Autonomic dysfunction”)],[TS = (“cardiovascular disease” AND “depression” AND “5-HT”)],[TS = (“cardiovascular disease” AND “depression” AND “ω-3 polyunsaturated fatty acids”)], [TS = (“cardiovascular disease” AND “depression” AND “Intestinal flora”)],[TS = (“cardiovascular disease” AND “depression” AND “Gene”)], [TS = (“cardiovascular disease” AND “depression” AND “MicroRNA”)]AND [language = (English)] AND [article type = (article AND reviews)] AND [Period = 2003 to December 2023)].

## 3. Epidemiology

In the 1930s, two longitudinal studies of depression found that depressed patients had a higher mortality rate from cardiovascular disease, but this relationship was not appreciated until the 1980s when interest in the role of depression in cardiovascular disease was raised. Since then, studies of cardiovascular disease with depression disease have been conducted around the world and have also confirmed the relationship between the two (2). Among Chinese adults, depression is associated with increased all-cause mortality and cardiovascular mortality, especially in men (14). Individuals with ischemic heart disease frequently feel depression, and compared to the general population, these individuals are more likely to develop atherosclerosis and have serious cardiovascular events. These two illnesses' fundamental pathophysiologic pathways are closely related (15). Up to 30% of heart failure patients suffer from depression, which is associated with a risk of heart failure, particularly in high-risk groups, and is strongly linked to poorer quality of life and clinical outcomes (16). In addition, a 3-fold increased fatalness of mental illness has been reported in patients after myocardial infarction (17). Patients with cardiovascular disease have a 26% increased risk of anxiety (18). In addition, mental disorder leads to higher all-cause mortality and cardiac mortality (19). In a Hong Kong-wide retrospective cohort study, long-term exposure to depression is associated with a significantly increased risk of cardiovascular disease. One thousand three hundred six (11.2%) of the 11,651 depressed patients have cardiovascular disease, and the results show that individuals with depression lasting 2–5 years and ≥6 years have a dramatically increased risk of cardiovascular disease compared to those who get depression within 1 year (20). Antidepressants have not been found to improve cardiovascular outcomes in randomized controlled studies in adults with depression and cardiovascular disease. Nevertheless, regardless of the strategy utilized, a reduction in depressed symptoms may result in a decrease in later cardiovascular events (21). A Mendelian randomization study suggests that genetic susceptibility to depression may have a positive causal effect on cardiovascular disease/myocardial infarction (CAD/MI) and that smoking and lipid levels may mediate the causative route (22).

Clinical studies have displayed that the prevalence of CVD patients with depression is high, ranging from 17% to 27% (23), the treatment rate is low, with less than 20% of patients with CVD being adequately treated for depressive symptoms (24). Risk factors include age, gender, social stress, medications, unhealthy lifestyle, and underlying disease (25). Myocardial infarction combined with depression is independently associated with about 2–4 times increased risk of subsequent cardiovascular events (26). A meta-analysis points out that appropriate exercise not only reduces mortality in patients with cardiovascular disease with depression diseases but also enhances the therapeutic effect of medications (27). In terms of prognosis, CVD patients with comorbid depression have worse prognostic performance (28), but a study reports that selective serotonin reuptake inhibitors (SSRIs) have a favorable impact on the prognosis of cardiovascular disease with depression diseases (29).

In conclusion, patients with cardiovascular disease with depression diseases, with high mortality, poor quality of life, and poor prognosis, are in urgent need of effective and stable treatment options.

## 4. Potential mechanism

There are many possible pathogenic mechanisms for depression in coronary artery disease, including the hypothalamic-pituitary-adrenal axis, genetic factors, autonomic dysfunction, 5-HT, microRNA, Omega-3 polyunsaturated fatty acids, and intestinal flora.

### 4.1. HPA axis

**Figure 1:**
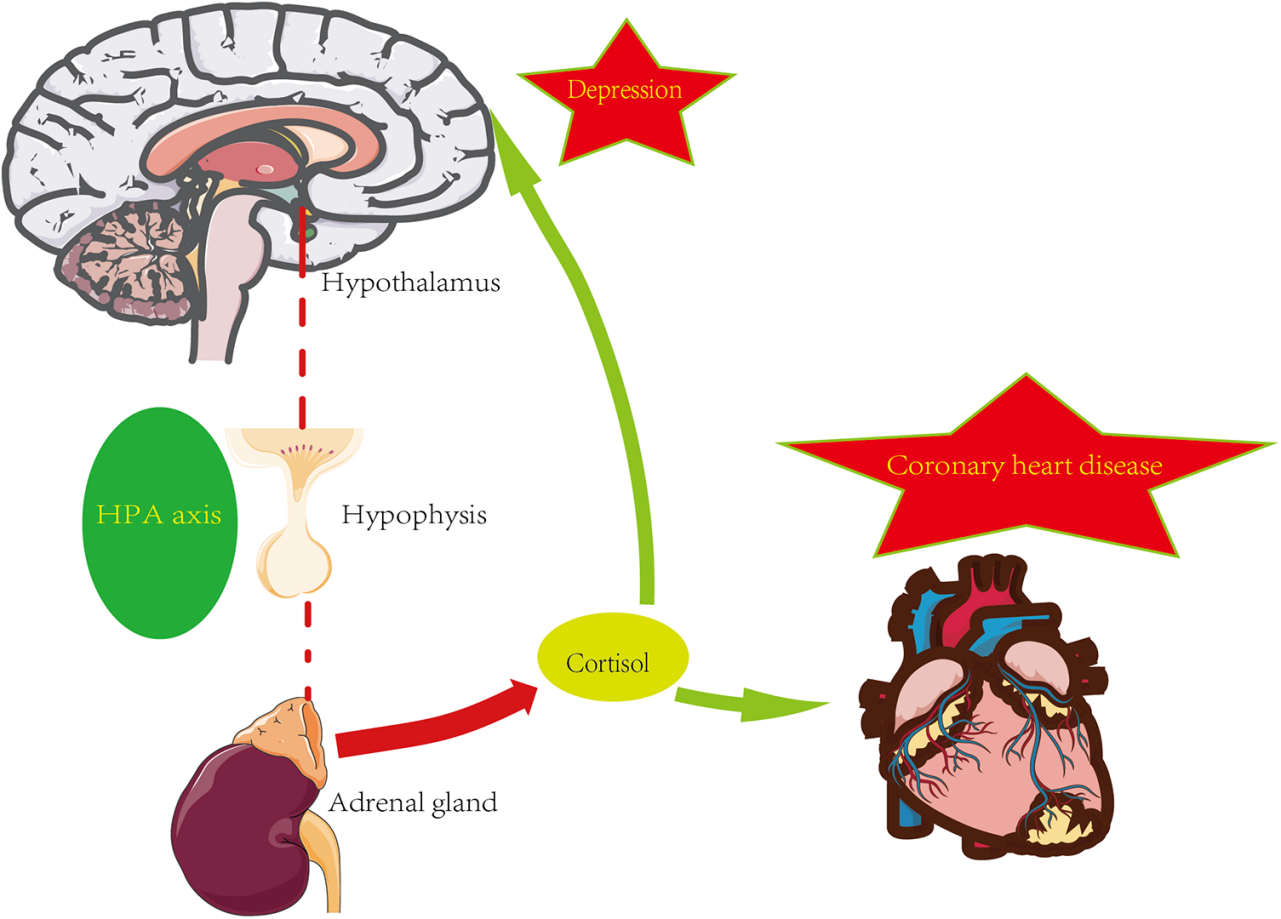
HPA axis and psycho-cardiology diseases.

The hypothalamic-pituitary-adrenal axis (HPA axis) is a complicated set of interactions between direct acts and feedback, and the HPA axis is an essential part of the neuroendocrine system. The maintenance of normal physiological events, including the stress response to internal and external stimuli, depends on proper HPA axis function (30). You can refer to the Figure 1 below.

Glucocorticoids play a key role in the development of coronary artery disease. Local effects in the vascular wall and myocardium are mediated by glucocorticoid receptors (MR and GR) and modified in a cell-specific pattern by 11β-hydroxysteroid dehydrogenase 1 (11b-HSD1) and 11β-hydroxysteroid dehydrogenase 2 (11b-HSD2). Some evidence suggests that these local effects not only induce cardiovascular risk factors during glucocorticoid overdose but also accelerate the onset and progression of atherosclerotic vascular disease (31). In several population-based cohort studies, activation of the HPA axis, accompanied by an increased rate of cortisol secretion and elevated morning plasma cortisol levels, not only predisposes to risk factors for coronary heart disease (e.g., hypertension, hyperglycemia, elevated triglycerides) but also exacerbates the development of atherosclerosis (32). Excessive cortisol can have a variety of negative effects on the heart, such as high blood pressure, trunk obesity, hyperinsulinemia, hyperglycemia, insulin resistance, and dyslipidemia. Some studies have shown that not only cortisol raises the risk of cardiovascular disease in Cushing's syndrome, but also in a broader range of conditions (33). It has also been shown that lower daily HPA axis activity appears to have a lower prognosis for cardiovascular health in healthy populations and cardiovascular patients by promoting a hypercoagulable condition. Thus, the development of atherosclerosis and its pathophysiology may be influenced by the downregulation of basal HPA axis activity (34).

Moreover, the HPA axis also plays a vital part in pathophysiological processes such as anxiety, depression, and cognitive dysfunction. For example, the glucocorticoid receptor (GR) is one of the most substantial brain receptors involved in the pathogenesis of depression and the mechanism of action of antipsychotic drugs, and the lack of “facilitation” of cortisol in the brain due to glucocorticoid resistance may be related to the pathogenesis of depression (35). A research comparing the hypothalamic-pituitary-adrenal (HPA) axis's performance in depressed and nondepressed people, cortisol disparities between the two groups are shown to be considerably larger. Additionally, significant variations in cortisol are seen in atypical, endogenous, depressive, and psychotic depression (36). In a study with major depressive disorder with psychotic major depression (PMD), non-psychotic major depression (NPMD), and healthy controls (HC), the results show that cognitive performance is negatively associated with high cortisol in all subjects, with PMD patients having higher cortisol than NPMD patients and HC patients (36, 37).

The HPA axis is also significantly altered in patients with coronary artery disease combined with depression. From a large randomized controlled multicenter pilot study, it was shown that in moderately depressed cardiovascular patients, anxious subjects exhibit lower waking and late-night cortisol levels, with a greater 30-min increase compared to non-anxious subjects (38). In a containing 83 participants research, patients with coronary artery disease with depression have lower plasma and salivary cortisol levels compared to patients without coronary artery disease, while the coronary artery disease depressed group also shows reduced glucocorticoid receptor expression and sensitivity (39).

### 4.2. Inflammation

**Figure 2:**
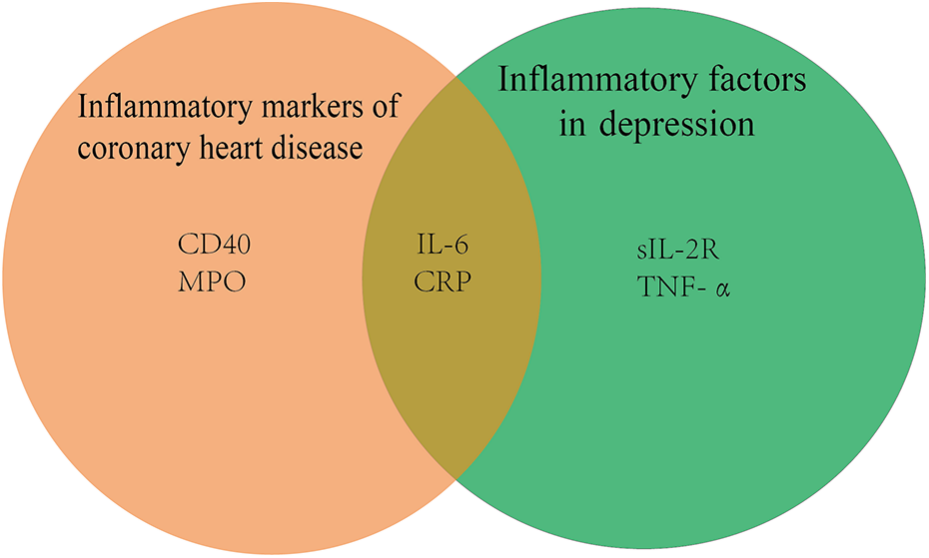
Inflammatory factors in cardiovascular disease with depression diseases CRP, c-reactive protein; MPO, myeloperoxidase; IL-6, interleukin 6; sIL-2R, soluble interleukin-2 receptor; TNF-α, tumor necrosis factor-α.

It is well known that coronary artery disease is characterized by the formation of arterial plaques, which are composed mainly of calcium, lipids, and inflammatory cells. Since inflammation is thought to play a significant role in the etiology of coronary artery disease, the levels of inflammatory biomarkers, such as IL-6, CD40,c-reactive protein (CRP), complement, and myeloperoxidase (MPO), can be used to gauge the severity and prognosis of coronary artery disease (40). Evidence suggests that TRAF6, a downstream target of CD40, is critical in the progression of atherogenesis, neointima development, and atherosclerosis when CD40 on macrophages is activated (41). Not only in cardiovascular disease but also in depression, there are changes in inflammatory factors, as shown in Figure 2 below.

A meta-analysis notes that serum sIL-2R, TNF-α, and IL-6 levels are significantly higher in patients with major depression than in healthy controls (42). There is proof that the tryptophan-kynurenine pathway's byproducts, including 3-hydroxykynurenine and quinolinic acid, are crucial in the neurodegenerative alterations of persistent severe depression (43). IL-6 may increase indoleamine-2,3-dioxygenase (IDO) activity, leading to activation of the kynurenine pathway, which triggers the production of the neurotoxic n-methyl-d-aspartate glutamate agonists quinolinic acid and 3-hydroxykynurenine, causing neurodegeneration and serious depression (44).

A study of 367,703 unrelated middle-aged subjects of European ancestry from UK Biobank suggests that co-morbidity of depression and coronary heart disease is mainly caused by common environmental factors. IL-6, CRP, and triglycerides (TG) may be causally related to depression and therefore may be targets for treatment and prevention of mental sickness (45). It has been suggested that the NLRP3 inflammasome may be activated by psychological stress, which releases IL-1, which may be involved in the pathophysiology of systemic disorders including diabetes and cardiovascular disease (46). In a cross-sectional study of depressed patients in mainland China, patients with major depression exhibit mild inflammation, elevated platelet and monocyte counts, elevated platelet/lymphocyte and monocyte/lymphocyte ratios, and upgraded systemic immune-inflammatory indices. In addition, monocyte count is the only factor significantly associated with the risk of coronary heart disease in patients with major depression (47).

### 4.3. Autonomic dysfunction

In altered mood states, autonomic alterations are commonly seen, and they appear to be the main physiologic mechanism connecting depression to several physical dysfunctions. Reduced (HRV) indices are a result of changes in autonomic nervous system function that encourage vagal withdrawal. Social stress in rodents triggers both depression-like behavior and cardiovascular changes consistent with excessive sympathetic drive, as well evidenced by reduced HRV and increased low frequency/high frequency (LF/HF) ratio (48). Researchers of depressed patients with coronary artery disease have found proof of autonomic nervous system (ANS) dysfunction, such as a rapid heartbeat and low variability of heart rate. Excessive heartbeat demonstrates body stressors, high ventricular repolarization variability, and low-pressure receptor susceptibility (49). Compared to non-depressed cardiac patients, relationships between inflammatory indices and autonomic function are greater in the depressed group (50). In addition, reduced HRV and stress reflex sensitivity are independent risk factors for cardiovascular disease. In addition, HRV and stress reflex sensitivity are also decreased in depressed patients (51). The study noted a linear relationship between the severity of depression and HRV index, with a significantly higher incidence of arrhythmias than in controls, especially supraventricular arrhythmias. The results suggest that depression is associated with cardiac autonomic nervous system dysfunction and that the severity of depression is related to the severity of this dysfunction. Depressed patients appear to be susceptible to early-onset atrial and/or ventricular disease (52). A random-effects ANOVA model shows a sex-dependent relationship between major depressive disorder and cardiac autonomic dysfunction, which provides an underlying explanation for the sex difference between depression symptoms and cardiovascular disease incidence (53).

### 4.4. 5-HT

**Figure 3:**
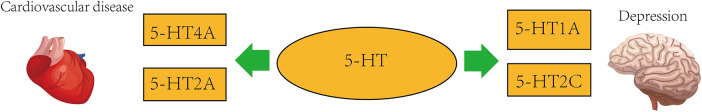
5-HT and psycho-cardiology diseases.

Depression is frequently brought on by stress, and the circadian system has a strong relationship with stress-sensitive neurotransmitter systems including the 5-hydroxytryptamine (5-HT) system. Some scientists obtained 5-HT nerve cell bodies and dendrites for the first time in 1967 and found the presence of 5-HT reuptake mechanisms in axons and nerve endings (54), antidepressants now have the potential to work by obstructing this mechanism. A biogenic amine called serotonin 5-hydroxytryptamine (5-HT) has a role in the pathophysiology of depression and cardiovascular disease as a neurotransmitter and peripheral hormone (55). It has been proposed that the 5-HT system is disturbed by stress, which interferes with circadian rhythms and makes people more susceptible to depression (56). Major depressive disorder (MDD) has been linked to the pathophysiology via the 5-hydroxytryptamine system. Additionally, it has been demonstrated that every antidepressant strategy increases 5-HT transmission in the brains of experimental animals (57). 5-HT1A receptor, located on the perinuclear and dendritic spines of 5-HT-containing neurons, is an auto-receptor and may limit the response of 5-HTergic neurons to afferent excitation. A 2010 study shows that following the administration of chronic selective 5-HT reuptake inhibitors (SSRIs) treatment, progressive downregulation or functional desensitization of 5-HT1A auto-receptors in the mid-suture dendrites after treatment with SSRIs attenuate their inhibitory effects on 5-HTergic neurons (58). Stroke is a serious disease worldwide. Although thrombolysis and thrombectomy have positive therapeutic effects, the recovery of neurological function after rescue is a worrying problem. Post-stroke depression (PSD) is one of the most common psychiatric problems among stroke survivors. In a prospective study of blood and urine samples from 28 patients (24 transient ischemic attacks and 4 hyperacute ischemic strokes) and 29 controls, 5-HT levels and 5-HT2 receptor levels were higher than in the control group. This suggests that 5-HT has an effect not only on psychiatric disorders but also on vascular diseases (59). According to one study, the clinical incidence of depression in patients after stroke ranges from 2% to 55% (60). Despite numerous clinical and experimental studies, the pathophysiological mechanisms of PSD are still far from clear. However, 5-HT transporters and their receptors play a crucial role in it (61). Antidepressant SSRIs are also used clinically as one of the drugs to treat PSD (62). For some information, you can refer to the Figure 3 below.

In the human heart, 5-HT4 receptor isoforms mediate the contractile, chronotropic, and proarrhythmic effects of 5-HT4, 5-HT receptor expression may be altered in cardiovascular disease (63). Combined treatment with 5-HT synthesis inhibitors and 5-HT2AR antagonists also synergistically inhibits atherosclerotic plaque formation and macrophage infiltration in ApoE-/- mice (64). Similar to hepatic steatosis, the pathogenesis of lipid atherosclerosis is connected with intracellular 5-HT2AR activation, 5-HT synthesis, and 5-HT degradation (64). In a rabbit model, the antagonist sarpogrelate blocks 5-HT2A receptors and may have antiproliferative effects on smooth muscle cells and macrophages by upregulating endothelial nitric oxide synthase (eNOS), thereby delaying the progression of atherosclerosis (65). In addition, selective serotonin reuptake inhibitor antidepressants (SSRIs) reduce cardiovascular morbidity and mortality, which may be related to serotonin and platelet abnormalities in depressed patients receiving effective treatment with SSRIs (66). The 5-HT1AR, 5-HT2AR chemoreceptor complex's inhibitory metabotropic receptor-receptor interactions may play a significant part in the regulation of mood, involving a reduction in post-conjugative 5-HT1AR prolamins signaling in the forebrain during 5-HT2AR prolamins activation, and disruption of the integrated the metabotropic interactions between receptors in the highly vulnerable 5-HT1A heteroreceptor complex may lead to major depression, and pathological blunting of the 5-HT2AR and, in particular, the OXTR protomer in the 5-HT2CR heteroreceptor complex may contribute to the emergence of psychiatric diseases like depression involving social-behavioral impairment (67). A study that included 300 patients with CAD (145 with acute coronary syndrome and 155 with stable coronary artery disease) noted a significantly higher incidence of major and minor adverse cardiac events in depressed cardiovascular patients. Serotonin receptor density was higher in patients with MDD and higher in patients with depressed cardiovascular disease, and future studies will require larger sample sizes (68). The S allele in the polymorphic region of the 5-hydroxytryptamine transporter (SERTs) gene has been shown to reduce transcription of the gene, thereby reducing 5-hydroxytryptamine reuptake, and some studies have reported that the S allele in the polymorphic region of the 5-hydroxytryptamine transporter gene is related with a higher risk of recurrent cardiac events in AMI patients, which is at least partially mediated by depressed symptoms in AMI patients (69).

### 4.5. MicroRNA

Small non-coding RNAs known as microRNAs (miRNAs) function as post-transcriptional regulators of gene expression. MicroRNAs are considered critical controllers of a variety of physiological and pathological processes associated with cardiovascular disease. Circulating miRNAs have been assessed as potential novel prognostic biomarkers for coronary artery disease, acute coronary syndromes, and acute myocardial infarction (70). Some studies suggest that miRNA and brain-derived neurotrophic factor (BDNF) may be involved in the process of depression in combination with essential hypertension (71). MiR-499 and miR-133a may be biomarkers of acute myocardial infarction (AMI), according to a meta-analysis of 19 studies that examine the specificity and sensitivity of miR-1, miR-133a, miR-208b, and miR-499 in AMI (72). In ischemia/reperfusion (I/R) rats, MiR-665 is elevated. While miR-665 knockdown has the opposite effect, miR-665 overexpression dramatically reduces LDH, CK-MB, TNF-, IL-6, and ROS concentrations and triggers apoptosis. By triggering Pak1/Akt signaling in myocardial infarction (MI), miR-665 knockdown reduces the buildup of ROS and apoptosis that are brought on by ischemia/reperfusion damage in cardiomyocytes (73). MiR-1202 is consistently dysregulated in postmortem brain tissue and blood of patients with major depression. The correlation between the predictive validity of miR-1202 on the response to antidepressant treatment in depressed patients and its peripheral variation and functional changes suggests that miR-1202 is involved in pathophysiological processes associated with depression (74). A finding suggests that miRNA analysis identifies hsa-miR-107 as an underlying biological link between APOE*ε*4, depressive symptoms, and cognitive impairment (75). Both the neurological and cardiovascular systems are affected by miR-132. Stress reduces BDNF levels. Low BDNF levels decrease activation of cyclic AMP effector element binding protein (CREB), leading to down-regulation of miR-132, which affects neuroplasticity and causes depression. In addition to raising glucocorticoid levels, stress also inhibits miR-132 through raising glucocorticoid levels. Through the autonomic nervous system and the HPA axis, miR-132 may have an impact on cardiovascular function (76). MiR-21 is a microRNA associated with cancer, development, and cardiovascular disease. In the study, human individuals with depression and alcoholism have considerably lower levels of miR-21 in the white matter close to the orbitofrontal cortex than non-psychotic controls. MiR-21 reduction is associated with some mRNAs for myelin proteins, the regulatory factor STAT3, and oligodendrocyte-associated transcription factors. Correlation suggests that miR-21 is involved in white matter alterations in depression and alcoholism (77). Results from a study in the Department of Cardiology at the First People's Hospital in Jining, China, in which peripheral blood samples were collected from 865 CAD patients, suggest that cardiovascular patients carrying the miR-146a *rs2910164 C* allele have a reduced risk of depression, an association that may be attributed to its ability to disrupt miR-146a expression, thereby increasing the expression of its target gene, *NOS1* (78). You can refer to Table 1 below.

**Table 1:** MicroRNA and cardiovascular disease with depression diseases.

Type	Effects on cardiovascular	Effects on depression	Reference
miR-1-3p	Express higher in patients with hypertension combined with depression	Express higher in patients with hypertension combined with depression	(57)
miR-133a	Key regulator of cardiac hypertrophy	–	(56)
miR-499	The expression level of diabetic cardiomyocytes was decreased	–	(58)
miR-665	Knockdown of miR-665 can prevent ROS accumulation and apoptosis induced by ischemia/reperfusion injury in cardiomyocytes	–	(59)
miR-1202	–	Down-regulated in the prefrontal cortex in depressed individuals	(60)
has-miR-107	–	The reduction of has-miR-107 may increase the risk of cognitive deficits	(61)
miR-132	Affects ANS and HPA axis	Decreased miR-132 expression, Inhibits BDNF-dependent neuronal function	(62)
miR-21	–	Involved in albumin changes in depression	(63)
miR-146a	–	Reducing the expression of miR-146a will lead to the expression of its target gene NOS1 and reduces the risk of depression in cardiovascular patients	(64)

### 4.6. ω-3 polyunsaturated fatty acids

Patients with coronary artery disease have lower concentrations of omega-3 fatty acids (FA). Supplementation with omega-3 fatty acids improves cardiovascular prognosis in patients with coronary heart disease and heart failure, and the GISSI-HF trial shows that supplementation with eicosapentaenoic acids (EPA) and docosahexaenoic acids (DHA) of omega-3 fatty acids, compared with placebo, improves survival in 6,975 patients with heart failure (HF) over a mean intervention period of 3.9 years, and reduce rehospitalization due to cardiovascular risk (79). In individuals with established coronary artery disease, omega-3 fatty acids have been demonstrated to dramatically lower the risk of sudden death owing to arrhythmias and all-cause mortality (80). Omega-3 fatty acids are also used in the treatment of hyperlipidemia and hypertension. The National Heart Association recommends two fish meals per week for people without a history of coronary heart disease and at least one fish meal per day for people with known heart illness. For cardioprotection, it is advised to consume around 1 g/day of EPA and DHA (81). Intake of n-3 polyunsaturated fatty acids (n-3 PUFA) boosts vascular and cardiac hemodynamics and may improve endothelial function, autonomic control, inflammation, arrhythmias, and thrombosis (82).

Depressed people have reduced amounts of n-3 PUFAs in their blood, red blood cells, adipose tissue, and brain tissue, according to studies looking into the n-3 PUFA status of depressed patients (83). Chronic dietary -3 PUFA shortage can alter the concentrations of ω-3 PUFA in the brain, causing changes in 5-hydroxytryptamine 2 (5-HT2) and dopamine 2 (D2) receptor density in the frontal cortex. The pathophysiology of depression is hypothesized to involve the activation of 5-HT2A/C receptors and downregulation of dopamine receptors (84). A 4-month randomized controlled study confirms the adjunctive mood stabilizing effect of ω-3-PUFA in bipolar disorder. ω-3 PUFA is more likely to result in better treatment outcomes than the placebo group in most other secondary indicators (85).

In this one study, severe mental illness patients' overall omega-3 index is below 4%, a range where the mortality risk from cardiovascular disease is high (86). In a study with a sample of 130 patients, adults aged 18–65 years with schizophrenia and depression supply blood samples and complete questionnaires and physiological exams. Results indicate that both populations have risk factors for metabolic syndrome and cardiovascular disease (87). Data from 44 subjects are analyzed in a study in which the docosahexaenoic acid (DHA), n-3 PUFA(N3), and n-6 PUFA(N6) to N3(N6/N3) ratios are lower in the moderately depressed group than in the non-depressed group (HAMD score <8), and the discrepancy in PUFA levels disappear in the mildly depressed group after the inclusion of patients with cardiovascular disease who are more heterogeneous in terms of depression. Thus, the role of n-3 PUFA is associated with depression in depressed patients if the depressive state is more strictly defined (88).

### 4.7. Intestinal flora

**Figure 4:**
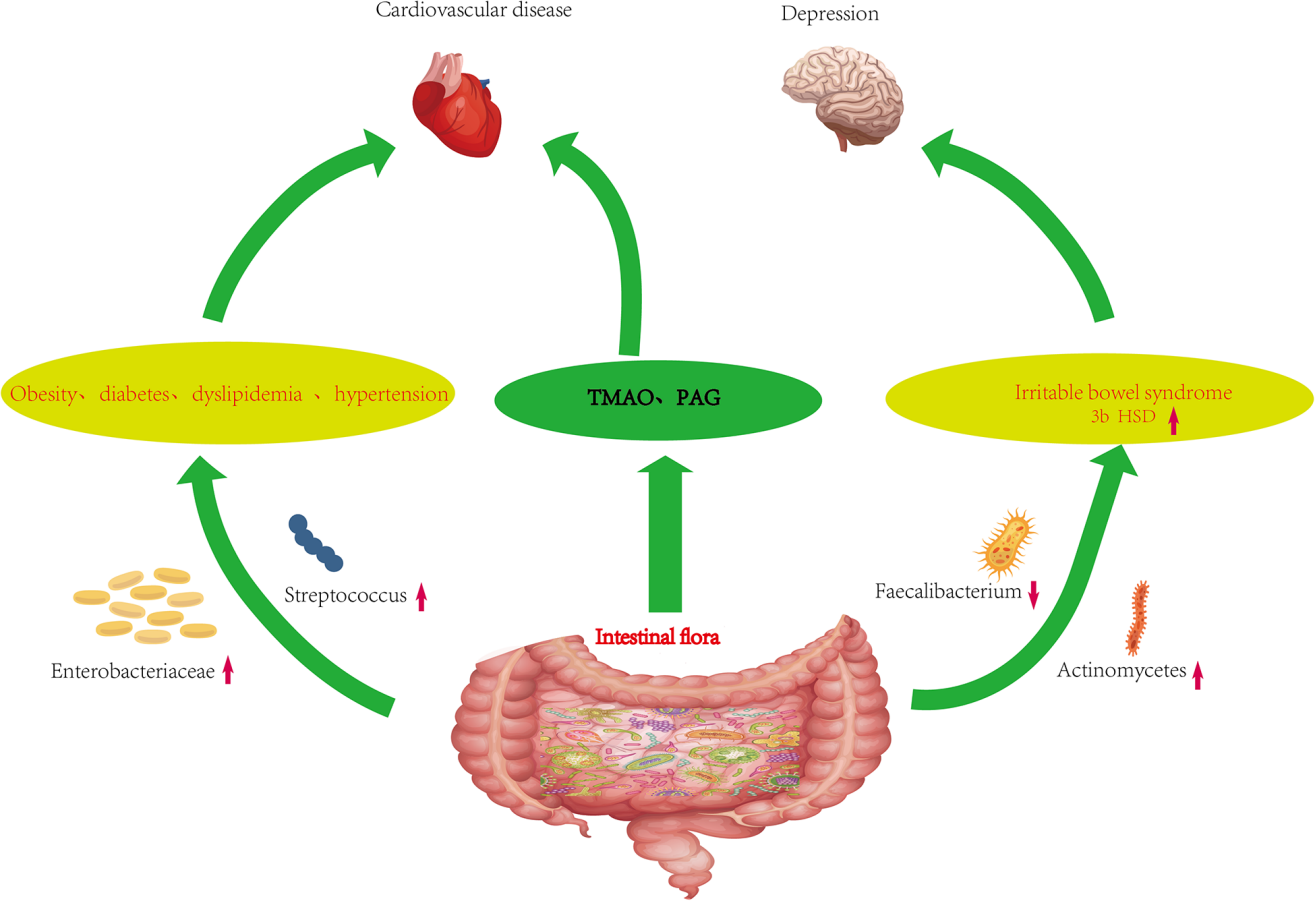
Intestinal flora and cardiovascular disease with depression diseases 3b HSD, 3b hydroxysteroid dehydrogenase; TMAO, trimethylamine N-oxide; PAG, phenylacetylglutamine.

Probiotic supplementation, fecal donation, and altering gut microbiota makeup might all be active study topics for the prevention and treatment of coronary heart disease (89). Several pathological conditions in the gastrointestinal tract may compromise the intestinal barrier, allowing bacteria and their metabolites to translocate to remote organs such as the heart, which may ultimately be in connection with the evolution of systemic inflammation and cardiovascular disease (90). Changes in fecal microbiota have been associated with many disease states, such as cardiovascular disease (CVD), phenylacetylglutamine (PAG) and trimethylamine N-oxide (TMAO) more recently are gut microbiota-dependent metabolites whose blood levels have been associated with CVD risk in large-scale clinical studies (91). A study including 218 patients with atherosclerotic cardiovascular disease and 187 healthy controls have a whole macrogenome association study of their feces. The gut microbiome in patients with atherosclerotic cardiovascular disease deviates from healthy status due to increased abundance of Enterobacteriaceae and Streptococcus spp (92). For some information, you can refer to the Figure 4 below.

Depression is strongly associated with variation, in the gastrointestinal microbiota. Depression has been linked to IBS (irritable bowel syndrome), which is a disorder where the gut microbiome is altered. Changes in the gut flora might worsen depression and trigger the stress response (93). Gut microbes can alleviate stress or depression-related symptoms by regulating brain function (94). Studies comparing the gut microbiota of the clinical group to the control group involved specific bacterial taxa. The most consistent finding is that participants with major depression/depressive disorder have a lower abundance of Phylum Bacteroides, Prevotella, Faecalibacterium, Coprococcus, and Sutterella and a higher abundance of Actinobacteria and Eggshells compared to controls (95). Administration of 3b hydroxysteroid dehydrogenase-producing E.coli to rats decreases their serum and brain testosterone levels and leads to depression-like behavior. Finally, 42.99% (46/107) of fecal samples from depressed patients contained 3b hydroxysteroid dehydrogenase (3b HSD) and 60.87% (28/46) expressed 3b HSD compared to 16.67% of participants without depression. These findings imply that the depressive symptoms brought on by testosterone breakdown may be related to 3b HSD expression by gut microorganisms (96).

### 4.8. Genetic factors

In one study, using pooled statistics from the largest genome-wide association study (GWAS) or GWAS meta-analysis of depression, both depression phenotypes were genetically associated with myocardial infarction by Mendelian randomization, and genetically, a doubling of the odds of depression was causally associated with an increased risk of coronary heart disease (22). Twin studies reveal an estimated 19% genetic correlation between depression and hypertension and about 42% genetic correlation between depression and heart disease. The genetic correlation between depressive symptoms and blood lipid levels ranged from 10% to 31% (97). Furthermore, although genetic variants associated with inflammation and serotonin may be associated with depression and cardiovascular disease, genetic changes associated with inflammation have been preliminarily examined for cardiovascular disease, whereas genetic variants in the serotonin system have been preliminarily examined for depression (98). In one study, using pooled statistics from genome-wide association studies of patients with depression and cardiovascular disease, Mendelian randomized analysis shows that genetic susceptibility to depression was associated with a higher risk of coronary heart disease and myocardial infarction (99). In one study, functional enrichment analysis suggests that platelet activation, chemokine signaling, and focal adhesion may be connected with heart failure combined with depression. The results show that a total of 5 pivotal genes, *CD83, CX3CR1, STAT4, COL1A2,* and *SH2D1B*, of which *STAT4* and *COL1A2* are important underlying the co-morbidity mechanism of heart failure and depression (100). A study based on aggregation statistics from genome-wide association studies of depression, heart disease, and nine cardiovascular risk factors shows that Confdr discovered 79 distinct loci linked to depressive symptoms, coronary artery disease, or cardiovascular risk factors. In addition, loci linked to an increased risk of depression are also linked to an increased risk of coronary artery disease and elevated levels of total cholesterol, low-density lipoprotein, and c-reactive protein. Six gene loci are linked to both mental illness and coronary artery disease (101).

## 5. Treatment

### 5.1. Drug treatment

The current pharmacological treatment for depression in combination with cardiovascular disease is mainly based on the addition of antidepressants to the cardiovascular prevention program. Drugs for secondary prevention of coronary heart disease mainly include two aspects: anti-myocardial ischemic drugs and drugs to improve prognosis and delay myocardial remodeling. The former mainly include nitrate drugs, β-blockers, calcium channel blockers, etc.; the latter mainly include antiplatelet drugs (Aspirin, Clopidogrel, Tegretol, Prasugrel, etc.), drugs to improve myocardial remodeling (β-blockers, ACEIs, and ARBs), and statins. There is therapeutic empirical evidence that the use of statins reduces the risk of depression in adults (102). A pooled analysis notes that vortioxetine is usually safe in MDD comorbid with cardiovascular disease, is well tolerated, and has no unexpected adverse events (103). Class A recommends medications for depression mainly include selective 5-hydroxytryptamine reuptake inhibitors (SSRI) (e.g., fluoxetine, paroxetine, fluvoxamine, sertraline, citalopram, escitalopram), selective 5-hydroxytryptamine and norepinephrine reuptake inhibitors (SNRI) (e.g., venlafaxine, duloxetine, milnacipran), norepinephrine and specific 5-hydroxytryptamine available antidepressant agents (NaSSA) (e.g., mirtazapine), and norepinephrine and dopamine reuptake inhibitors (NDRI) (e.g., bupropion). SSRIs, SNRIs, and some newer antidepressants (e.g., melatonin MT1/MT2 receptor agonists and 5-hydroxytryptamine receptor antagonists: agomelatine) are also classified as A-recommended for their safety and tolerability advantages over traditional tricyclics and monoamine oxidase inhibitors, but the evidence for their use in cardiovascular disease is mixed. A randomized controlled trial shows that 12 months of treatment with escitalopram prevents depression in post-ACS patients (104). One prospective multicenter study shows that mirtazapine appears to be safe in the treatment of depression after myocardial infarction (105). For some information, you can refer to the Table 2 below. In addition to this, traditional Chinese medicine (TCM) has revealed its unique advantages in the treatment of cardiovascular disease with depression diseases with its multi-targets and low rate of adverse effects. Shuangxinfang can alleviate cardiac impairment and improve depressive-like behavior by affecting S100A9-mediated macrophage/microglia inflammation (106). Chaihujialonggumuli granules can reduce inflammation and treat coexisting angst after myocardial infarction by inhibiting CXCR4/NF-κB/GSDMD signaling (107). Yixin Ningshen Tablet may alleviate myocardial infarction by enhancing myocardial energy metabolism. By reducing inflammation and improving the availability of monoamine neurotransmitters, it may also help treat depression (108).

**Table 2:** Drugs to treat cardiovascular disease and depression.

Drug class	Representative	Site of action	Cardiovascular effects	Depression effects
Nitrates	Nitroglycerin, isosorbide nitrate	Release nitric oxide (NO), relaxes vascular smooth muscle dilates coronary vessels	Improves myocardial ischemia	–
β-blockers	Metoprolol, labetalol	Binds to β-adrenergic receptors inhibits sympathetic nerves	Reduces cardiac load	–
Calcium channel blockers	Nifedipine, amlodipine	Act on vascular smooth muscle Ca2 + channels, inhibit Ca2 inward flow	Reduce myocardial contractility and slow heart rate	–
Antiplatelets	Aspirin, clopidogrel	Aspirin is a cyclooxygenase inhibitor, and clopidogrel acts on adenosine diphosphate	Prevent platelet aggregation and thrombosis	–
ACEI	Captopril, enalapril	Inhibit the conversion of angiotensin I to angiotensin II dilates blood vessels	Lowers blood pressure	Therapeutic effect
ARB	Losartan, valsartan	Selective blockade of angiotensin II receptors dilates blood vessels	Lowers blood pressure	–
Statins	Atorvastatin, simvastatin	Selective inhibition of HMG-CoA reductase	Lower cholesterol and LDL	Therapeutic effects
Monoamine oxidase inhibitors (MAOIs)	Moclobemide	Inhibits the activity of monoamine oxidase *in vivo* regulates blood pressure Increases the level of catecholamines at the synaptic site	Resulting in an antidepressant effect	
Tricyclic antidepressants (TCAs)	Promethazine	Inhibits central nervous uptake of norepinephrine (NE) and 5-hydroxytryptamine (5-HT) reuptake	Cardiotoxicity	Increases synaptic gap monoamine transmitter concentrations
Selective 5-hydroxytryptamine reuptake inhibitors (SSRIs)	Fluoxetine, citalopram	Block 5-hydroxytryptamine transporters in neurons	Safe	Inhibits reuptake of 5-HT increases concentration of 5-HT in the synaptic gap
Selective 5-hydroxytryptamine and norepinephrine reuptake inhibitors (SNRIs)	Venlafaxine duloxetine	Selective action on 5-HT receptors inhibits the reuptake of NE	Risk of increased blood pressure	Increased concentration of monoamine transmitters in the synaptic gap
Norepinephrine and specific 5-hydroxytryptamine antidepressants (NASSAs)	Mirtazapine	Acts at central presynaptic α2 receptors, interacts safely with central 5-hydroxytryptamine (5-HT2, 5-HT3) receptors	–	Enhances adrenergic neurotransmission, regulates 5-HT function
Norepinephrine and dopamine reuptake inhibitors (NDRIs)	Bupropion	Inhibits norepinephrine and dopamine reuptake uptake	–	Selectively inhibits norepinephrine and dopamine uptake

### 5.2. Non-pharmacologic treatments

In addition to pharmacologic treatments, some studies have used psychotherapy to manage depression in cardiac patients. One approach to support depression in CVD patients is the use of traditional face-to-face cognitive behavioral therapy (CBT), which has been demonstrated to be beneficial in CVD patients and has been suggested as a first-line therapy for depression (109). In addition to this, patients with cardiovascular disease (CVD) have good short-term benefits from depression through internet-based cognitive behavioral treatment (ICBT) (110). Care management is likewise important in the treatment of bipolar heart disease, with a prospective randomized trial specifying that considerable improvements in the outcomes of mental health, as well as depression, are associated with fewer cardiac symptoms after 12 weeks of collaborative care (111). Furthermore, in patients with myocardial infarction, exercise-based cardiac rehabilitation has been shown to alleviate anxiety and depressive symptoms (112). As medicine advances, cognitive therapy, behavioral therapy, and collaborative care will become more involved in the treatment of patients with dual heart disease.

## 6. Discussion

This paper begins with the epidemiology of depression in combination with cardiovascular disease and addresses potential pathogenic mechanisms in turn. The first place is currently placed in the pituitary-adrenal axis and a key substance, corticosterone, is noted. Corticosterone, a glucocorticoid hormone, acts in the MR and GR and has a crucial role in cardiomyocyte damage and repair; meanwhile, clinical studies have suggested that levels of corticosterone vary greatly between depressed and non-depressed patients. Despite the lack of studies at the molecular level, we may venture to speculate that corticosterone is likely the key molecule linking cardiovascular disease and depression. The inflammatory response is a defense response to stimuli in the body. In the serum of patients with cardiovascular disease and depression, the levels of IL6 and CRP are higher than those of normal people, and more critically, IL6 and CRP can link the brain and heart through blood circulation. Heart rate variability (HRV) reflects autonomic function and diminished HRV is an independent risk factor for cardiovascular disease, while HRV index is positively correlated with the severity of depression. Autonomic dysfunction is likely to contribute to the pathogenesis of depression in patients with coronary heart disease combined with depression. 5-HT is a neurotransmitter as well as a peripheral hormone. As a neurotransmitter, its changes reflect the state of nerve cells; as a peripheral hormone, it is associated with coronary atheromatous plaque formation and macrophage infiltration. Circulating microRNAs are key regulatory molecules that modulate pathophysiological processes and may be associated with cardiovascular disease comorbid with depression. omega-3 fatty acids have been shown to significantly reduce the risk of sudden death and all-cause mortality in patients with cardiovascular disease, and coincidentally, patients with depression also have a low index of omega-3 polyunsaturated fatty acids. Changes in gut flora lead to corresponding changes in metabolites. They affect the development of heart disease and depression through different pathways. By way of summary, it was found that studies have reported six genetic loci associated with depression and coronary artery disease, and a genetic correlation between depression and heart disease of about 42%, among other information. Although basic research is still lacking, I believe that the pathogenesis of cardiovascular comorbid depression will eventually come to light as experts continue to focus on it.

Treatment is divided into pharmacological and non-pharmacological treatments. The current strategy of pharmacological treatment uses the addition of antidepressants on top of cardiovascular prevention programs. With the increasing abundance of clinical data, traditional Chinese medicine is also slowly entering the clinician's field of vision. Non-pharmacological treatment focuses on clinical care, and cognitive behavioral therapy, currently used as a first-line treatment for depression, is also effective in patients with cardiovascular disease-combined depression.

## 7. Limitation

First of all, this review paper is based on my analysis and summary of all the cited literature. Although I tried to be objective in the analysis process, it is still highly subjective, and all the findings are based on some views derived from personal summaries. Second, several pathogenic mechanisms have been hypothesized to support the relationship between cardiovascular disease and depression. However, none has been shown to account for a small fraction of the risk, nor has it gone down to the level of molecular mechanisms, although this may be related to insufficient basic research, which still needs to be dug deeper. Some potential intermediate molecules, such as corticosterone, 5-HT, IL6, CRP, and omega-3 polyunsaturated fatty acids, have been identified, but there is a lack of exploration of the upstream and downstream of these molecules and a comprehensive understanding of this pathogenesis. Autonomic dysfunction, circulating microRNA, intestinal flora, genetics, and other research directions have been found, but there is still a long way to go for in-depth research. Depression and cardiovascular disease are closely related, but this article does not discuss the chemical changes and physiological structures behind the interaction of the two diseases. Finally, in terms of drug therapy, there is no clear target for the treatment of depression combined with cardiovascular disease. The effectiveness of TCM in the treatment of cardiovascular diseases combined with depression has been confirmed in Asia, but worldwide clinical data are lacking. Subsequent studies are expected to support the authors' conclusions.

## 8. Future prospects

Clinical treatment of cardiovascular disease drugs combined with antidepressants for the treatment of cardiovascular disease comorbid with depression, how to choose the appropriate drugs, and how to evaluate the benefit of one drug for two diseases, looking forward to more clinical studies in the future. Chinese medicine is gradually recognized for its multi-targeting and low adverse reaction rate and is expected to become the mainstay of treatment for multi-systemic diseases in the future.

Pathogenesis, the pathogenesis of cardiovascular disease complicated with depression is complex, there is no clear conclusion, and the potential pathogenesis needs more basic research to verify. Focusing on the alterations of corticosterone, 5-HT, IL6, CRP, and ω-3 polyunsaturated fatty acids and their upstream and downstream changes, these are the directions worth exploring. New ideas on the pathogenesis of cardiovascular disease-combined depression focus on gut flora and Micro RNA, which have an impact on both depression and cardiovascular disease, and it is worthwhile to think about how to find the substances that play a central role. The development of depression in combination with cardiovascular disease may involve organ communication, and the use of RNA sequencing and gene enrichment may allow us to understand the pathophysiological changes in one organ when the pathophysiology of the other organ is altered.

A correct understanding of the pathogenesis of double heart disease is very important for identifying and treating the disease, which will reduce the morbidity and mortality of cardiovascular disease, improve the symptoms of patients with anxiety depression, and other mental diseases, and improve the quality of life of patients.

## 9. Conclusion

Clinical and related research data have shown that cardiovascular disease and depression are highly correlated, the incidence of cardiovascular disease combined with depression is higher 17%–27%, and the cure rate is poor, less than 20%. The potential pathogenesis of cardiovascular diseases combined with depression includes HPA axis disorder, inflammation, genetic factors, autonomic nervous dysfunction, 5-HT disorder, microRNA disorder, Omega-3 polyunsaturated fatty acid disorder, intestinal flora disorder, etc. In addition, corticosterone, 5-HT, Omega-3 fatty acids, CRP, IL-6, miR-132, miR-1-3p, and other substances may play an intermediate role in the physiological and pathological process of cardiovascular diseases complicated with depression. Treatment is divided into drug therapy and non-drug therapy. The current drug treatment strategy is to add antidepressants to cardiovascular prevention programs. With increasingly rich clinical data, traditional Chinese medicine is slowly entering the field of vision of clinicians. Non-pharmacological treatments focus on clinical care and cognitive behavior.

## References

[1] You Y Shou X Zhang X Fan S Chai R Xue W Psycho-cardiological disease: a bibliometric review from 2001 to 2021. Front Cardiovasc Med. (2022) 9:890329. 10.3389/fcvm.2022.890329 35571163 PMC9099051

[2] Carney RM Freedland KE. Depression and coronary heart disease. Nat Rev Cardiol. (2017) 14(3):145–55. 10.1038/nrcardio.2016.181 27853162

[3] Daniel M Agewall S Berglund F Caidahl K Collste O Ekenbäck C Prevalence of anxiety and depression symptoms in patients with myocardial infarction with non-obstructive coronary arteries. Am J Med. (2018) 131(9):1118–24. 10.1016/j.amjmed.2018.04.040 29859805

[4] Fox KAA Metra M Morais J Atar D. The myth of “stable” coronary artery disease. Nat Rev Cardiol. (2020) 17(1):9–21. 10.1038/s41569-019-0233-y 31358978

[5] Harsanyi S Kupcova I Danisovic L Klein M. Selected biomarkers of depression: what are the effects of cytokines and inflammation? Int J Mol Sci. (2022) 24(1):578. 10.3390/ijms24010578 36614020 PMC9820159

[6] Zhang Y Chen Y Ma L. Depression and cardiovascular disease in elderly: current understanding. J Clin Neurosci. (2018) 47:1–5. 10.1016/j.jocn.2017.09.022 29066229

[7] Dickens C. Depression in people with coronary heart disease: prognostic significance and mechanisms. Curr Cardiol Rep. (2015) 17(10):83. 10.1007/s11886-015-0640-6 26277367

[8] Thombs BD Bass EB Ford DE Stewart KJ Tsilidis KK Patel U Prevalence of depression in survivors of acute myocardial infarction. J Gen Intern Med. (2006) 21(1):30–8. 10.1111/j.1525-1497.2005.00269.x 16423120 PMC1484630

[9] Chen X Zeng M Chen C Zhu D Chen L Jiang Z. Efficacy of psycho-cardiology therapy in patients with acute myocardial infarction complicated with mild anxiety and depression. Front Cardiovasc Med. (2022) 9:1031255. 10.3389/fcvm.2022.1031255 36776943 PMC9909477

[10] Jiang W Velazquez EJ Kuchibhatla M Samad Z Boyle SH Kuhn C Effect of escitalopram on mental stress-induced myocardial ischemia: results of the remit trial. JAMA. (2013) 309(20):2139–49. 10.1001/jama.2013.5566 23695483 PMC4378823

[11] Zambrano J Celano CM Januzzi JL Massey CN Chung WJ Millstein RA Psychiatric and psychological interventions for depression in patients with heart disease: a scoping review. J Am Heart Assoc. (2020) 9(22):e018686. 10.1161/jaha.120.018686 33164638 PMC7763728

[12] Yang Y Huang T Zhang H Li X Shi S Tian X Formononetin improves cardiac function and depressive behaviours in myocardial infarction with depression by targeting gsk-3β to regulate macrophage/microglial polarization. Phytomedicine. (2023) 109:154602. 10.1016/j.phymed.2022.154602 36610138

[13] Qian Y Chen L Gao B Ye X. Sestrin2 levels in patients with anxiety and depression myocardial infarction was up-regulated and suppressed inflammation and ferroptosis by Lkb1-mediated ampk activation. Clin Exp Hypertens. (2023) 45(1):2205049. 10.1080/10641963.2023.2205049 37183711

[14] Meng R Yu C Liu N He M Lv J Guo Y Association of depression with all-cause and cardiovascular disease mortality among adults in China. JAMA Netw Open. (2020) 3(2):e1921043. 10.1001/jamanetworkopen.2019.21043 32049295 PMC7212017

[15] Pivato CA Chandiramani R Petrovic M Nicolas J Spirito A Cao D Depression and ischemic heart disease. Int J Cardiol. (2022) 364:9–15. 10.1016/j.ijcard.2022.05.056 35643217

[16] Sbolli M Fiuzat M Cani D O’Connor CM. Depression and heart failure: the lonely comorbidity. Eur J Heart Fail. (2020) 22(11):2007–17. 10.1002/ejhf.1865 32468714

[17] Lippi G Montagnana M Favaloro EJ Franchini M. Mental depression and cardiovascular disease: a multifaceted, bidirectional association. Semin Thromb Hemost. (2009) 35(3):325–36. 10.1055/s-0029-1222611 19452408

[18] Shang X Peng W Hill E Szoeke C He M Zhang L. Incidence of medication-treated depression and anxiety associated with long-term cancer, cardiovascular disease, diabetes and osteoarthritis in community-dwelling women and men. EClinicalMedicine. (2019) 15:23–32. 10.1016/j.eclinm.2019.08.010 31709411 PMC6833452

[19] Zhang L Bao Y Tao S Zhao Y Liu M. The association between cardiovascular drugs and depression/anxiety in patients with cardiovascular disease: a meta-analysis. Pharmacol Res. (2022) 175:106024. 10.1016/j.phrs.2021.106024 34890773

[20] Zhang Y Li X Chan VKY Luo H Chan SSM Wong GHY Depression duration and risk of incident cardiovascular disease: a population-based six-year cohort study. J Affect Disord. (2022) 305:188–95. 10.1016/j.jad.2022.03.005 35283180

[21] Shiga T. Depression and cardiovascular diseases. J Cardiol. (2023) 81(5):485–90. 10.1016/j.jjcc.2022.11.010 36410589

[22] Li GH Cheung CL Chung AK Cheung BM Wong IC Fok MLY Evaluation of bi-directional causal association between depression and cardiovascular diseases: a Mendelian randomization study. Psychol Med. (2022) 52(9):1765–76. 10.1017/s0033291720003566 33032663

[23] Lichtman JH Froelicher ES Blumenthal JA Carney RM Doering LV Frasure-Smith N Depression as a risk factor for poor prognosis among patients with acute coronary syndrome: systematic review and recommendations: a scientific statement from the American heart association. Circulation. (2014) 129(12):1350–69. 10.1161/cir.0000000000000019 24566200

[24] Huffman JC Smith FA Blais MA Beiser ME Januzzi JL Fricchione GL. Recognition and treatment of depression and anxiety in patients with acute myocardial infarction. Am J Cardiol. (2006) 98(3):319–24. 10.1016/j.amjcard.2006.02.033 16860016

[25] Carney RM Freedland KE Steinmeyer BC Rich MW. Symptoms that remain after depression treatment in patients with coronary heart disease. J Psychosom Res. (2023) 165:111122. 10.1016/j.jpsychores.2022.111122 36608512 PMC10249067

[26] Meijer A Conradi HJ Bos EH Thombs BD van Melle JP de Jonge P. Prognostic association of depression following myocardial infarction with mortality and cardiovascular events: a meta-analysis of 25 years of research. Gen Hosp Psychiatry. (2011) 33(3):203–16. 10.1016/j.genhosppsych.2011.02.007 21601716

[27] Knapen J Vancampfort D Moriën Y Marchal Y. Exercise therapy improves both mental and physical health in patients with Major depression. Disabil Rehabil. (2015) 37(16):1490–5. 10.3109/09638288.2014.972579 25342564

[28] Blatch Armon D Buhayer A Dobretz K Meinlschmidt G Battegay E. Clinical practice guidelines for cardiovascular disease: how is depression addressed? Protocol for a systematic review. BMJ Open. (2023) 13(5):e071940. 10.1136/bmjopen-2023-071940 37130663 PMC10163515

[29] Akosile W Tiyatiye B Colquhoun D Young R. Management of depression in patients with coronary artery disease: a systematic review. Asian J Psychiatr. (2023) 83:103534. 10.1016/j.ajp.2023.103534 36871435

[30] Karaca Z Grossman A Kelestimur F. Investigation of the hypothalamo-pituitary-adrenal (HPA) axis: a contemporary synthesis. Rev Endocr Metab Disord. (2021) 22(2):179–204. 10.1007/s11154-020-09611-3 33770352

[31] Walker BR. Glucocorticoids and cardiovascular disease. Eur J Endocrinol. (2007) 157(5):545–59. 10.1530/eje-07-0455 17984234

[32] Walker BR. Cortisol–cause and cure for metabolic syndrome? Diabet Med. (2006) 23(12):1281–8. 10.1111/j.1464-5491.2006.01998.x 17116176

[33] Whitworth JA Williamson PM Mangos G Kelly JJ. Cardiovascular consequences of cortisol excess. Vasc Health Risk Manag. (2005) 1(4):291–9. 10.2147/vhrm.2005.1.4.291 17315601 PMC1993964

[34] Degroote C von Känel R Thomas L Zuccarella-Hackl C Messerli-Bürgy N Saner H Lower diurnal HPA-axis activity in male hypertensive and coronary heart disease patients predicts future CHD risk. Front Endocrinol (Lausanne*)* (2023) 14:1080938. 10.3389/fendo.2023.1080938 36967749 PMC10036761

[35] Pariante CM. The glucocorticoid receptor: part of the solution or part of the problem? J Psychopharmacol. (2006) 20(4 Suppl):79–84. 10.1177/1359786806066063 16785275

[36] Stetler C Miller GE. Depression and hypothalamic-pituitary-adrenal activation: a quantitative summary of four decades of research. Psychosom Med. (2011) 73(2):114–26. 10.1097/PSY.0b013e31820ad12b 21257974

[37] Keller J Gomez R Williams G Lembke A Lazzeroni L Murphy GM Jr. HPA axis in major depression: cortisol, clinical symptomatology and genetic variation predict cognition. Mol Psychiatry (2017) 22(4):527–36. 10.1038/mp.2016.120 27528460 PMC5313380

[38] Weber C Fangauf SV Michal M Ronel J Herrmann-Lingen C Ladwig KH Cortisol awakening reaction and anxiety in depressed coronary artery disease patients. J Clin Med. (2022) 11(2):374. 10.3390/jcm11020374 35054071 PMC8779785

[39] Nikkheslat N Zunszain PA Horowitz MA Barbosa IG Parker JA Myint AM Insufficient glucocorticoid signaling and elevated inflammation in coronary heart disease patients with comorbid depression. Brain Behav Immun. (2015) 48:8–18. 10.1016/j.bbi.2015.02.002 25683698

[40] Li H Sun K Zhao R Hu J Hao Z Wang F Inflammatory biomarkers of coronary heart disease. Front Biosci (Schol Ed). (2018) 10(1):185–96. 10.2741/s508 28930526

[41] Jansen MF Hollander MR van Royen N Horrevoets AJ Lutgens E. CD40 in coronary artery disease: a matter of macrophages? Basic Res Cardiol. (2016) 111(4):38. 10.1007/s00395-016-0554-5 27146510 PMC4856717

[42] Liu Y Ho RC Mak A. Interleukin (IL)-6, tumour necrosis factor alpha (TNF-α) and soluble interleukin-2 receptors (sIL-2r) are elevated in patients with major depressive disorder: a meta-analysis and meta-regression. J Affect Disord. (2012) 139(3):230–9. 10.1016/j.jad.2011.08.003 21872339

[43] Leonard BE. Inflammation and depression: a causal or coincidental link to the pathophysiology? Acta Neuropsychiatr. (2018) 30(1):1–16. 10.1017/neu.2016.69 28112061

[44] Ting EY Yang AC Tsai SJ. Role of interleukin-6 in depressive disorder. Int J Mol Sci. (2020) 21(6):2193. 10.3390/ijms21062194 32235786 PMC7139933

[45] Khandaker GM Zuber V Rees JMB Carvalho L Mason AM Foley CN Shared mechanisms between coronary heart disease and depression: findings from a large UK general population-based cohort. Mol Psychiatry. (2020) 25(7):1477–86. 10.1038/s41380-019-0395-3 30886334 PMC7303009

[46] Alcocer-Gómez E Cordero MD. NLRP_3_ inflammasome: common nexus between depression and cardiovascular diseases. Nat Rev Cardiol. (2017) 14(2):124. 10.1038/nrcardio.2016.214 28054580

[47] Zhou L Ma X Wang W. Inflammation and coronary heart disease risk in patients with depression in China mainland: a cross-sectional study. Neuropsychiatr Dis Treat. (2020) 16:81–6. 10.2147/ndt.S216389 32021201 PMC6957094

[48] Sgoifo A Carnevali L Alfonso Mde L Amore M. Autonomic dysfunction and heart rate variability in depression. Stress. (2015) 18(3):343–52. 10.3109/10253890.2015.1045868 26004818

[49] Carney RM Freedland KE Veith RC. Depression, the autonomic nervous system, and coronary heart disease. Psychosom Med. (2005) 67(Suppl 1):S29–33. 10.1097/01.psy.0000162254.61556.d5 15953797

[50] Frasure-Smith N Lespérance F Irwin MR Talajic M Pollock BG. The relationships among heart rate variability, inflammatory markers and depression in coronary heart disease patients. Brain Behav Immun. (2009) 23(8):1140–7. 10.1016/j.bbi.2009.07.005 19635552

[51] Pinter A Szatmari S Jr. Horvath T Penzlin AI Barlinn K Siepmann M Cardiac dysautonomia in depression—heart rate variability biofeedback as a potential add-on therapy. Neuropsychiatr Dis Treat (2019) 15:1287–310. 10.2147/ndt.S200360 31190834 PMC6529729

[52] Wang Y Zhao X O’Neil A Turner A Liu X Berk M. Altered cardiac autonomic nervous function in depression. BMC Psychiatry. (2013) 13:187. 10.1186/1471-244x-13-187 23842138 PMC3710510

[53] Garcia RG Zarruk JG Guzman JC Barrera C Pinzon A Trillos E Sex differences in cardiac autonomic function of depressed young adults. Biol Psychol. (2012) 90(3):179–85. 10.1016/j.biopsycho.2012.03.016 22504296

[54] Fuxe K Ungerstedt U. Localization of 5-hydroxytryptamine uptake in rat brain after intraventricular injection. J Pharm Pharmacol. (1967) 19(5):335–7. 10.1111/j.2042-7158.1967.tb08097.x 4382408

[55] Rieder M Gauchel N Bode C Duerschmied D. Serotonin: a platelet hormone modulating cardiovascular disease. J Thromb Thrombolysis. (2021) 52(1):42–7. 10.1007/s11239-020-02331-0 33155668 PMC8282555

[56] Daut RA Fonken LK. Circadian regulation of depression: a role for serotonin. Front Neuroendocrinol. (2019) 54:100746. 10.1016/j.yfrne.2019.04.003 31002895 PMC9826732

[57] Blier P El Mansari M. Serotonin and beyond: therapeutics for major depression. Philos Trans R Soc Lond B Biol Sci. (2013) 368(1615):20120536. 10.1098/rstb.2012.0536 23440470 PMC3638389

[58] Popa D Cerdan J Repérant C Guiard BP Guilloux JP David DJ A longitudinal study of 5-HT outflow during chronic fluoxetine treatment using a new technique of chronic microdialysis in a highly emotional mouse strain. Eur J Pharmacol. (2010) 628(1–3):83–90. 10.1016/j.ejphar.2009.11.037 19944680

[59] Saccaro LF Pico F Chadenat ML Richard O Launay JM Bastenaire B Platelet, plasma, urinary tryptophan-serotonin-kynurenine axis markers in hyperacute brain ischemia patients: a prospective study. Front Neurol. (2021) 12:782317. 10.3389/fneur.2021.782317 35087467 PMC8787359

[60] Jørgensen TSH Wium-Andersen IK Wium-Andersen MK Jørgensen MB Prescott E Maartensson S Incidence of depression after stroke, and associated risk factors and mortality outcomes, in a large cohort of Danish patients. JAMA Psychiatry. (2016) 73(10):1032–40. 10.1001/jamapsychiatry.2016.1932 27603000

[61] J.Pharmthera.2017.11.005 (1).Pdf. 10.1016/j.pharmthera.2017.11.005

[62] Robinson RG Jorge RE Starkstein SE. Poststroke depression: an update. J Neuropsychiatry Clin Neurosci. (2023). 10.1176/appi.neuropsych.21090231 37559511

[63] Neumann J Hofmann B Dhein S Gergs U. Cardiac roles of serotonin (5-HT) and 5-HT-receptors in health and disease. Int J Mol Sci. (2023) 24(5):4765. 10.3390/ijms24054765 36902195 PMC10003731

[64] Ma Y Liang X Li C Li R Tong X Zhang R 5-HT(2A) Receptor and 5-HT degradation play a crucial role in atherosclerosis by modulating macrophage foam cell formation, vascular endothelial cell inflammation, and hepatic steatosis. J Atheroscler Thromb. (2022) 29(3):322–36. 10.5551/jat.58305 33536397 PMC8894120

[65] Hayashi T Sumi D Matsui-Hirai H Fukatsu A Arockia Rani PJ Kano H Sarpogrelate HCI, a selective 5-HT2A antagonist, retards the progression of atherosclerosis through a novel mechanism. Atherosclerosis. (2003) 168(1):23–31. 10.1016/s0021-9150(03)00054-6 12732383

[66] Wozniak G Toska A Saridi M Mouzas O. Serotonin reuptake inhibitor antidepressants (SSRIs) against atherosclerosis. Med Sci Monit. (2011) 17(9):Ra205-14. 10.12659/msm.881924 21873959 PMC3560505

[67] Borroto-Escuela DO Ambrogini P Chruścicka B Lindskog M Crespo-Ramirez M Hernández-Mondragón JC The role of central serotonin neurons and 5-HT heteroreceptor complexes in the pathophysiology of depression: a historical perspective and future prospects. Int J Mol Sci. (2021) 22(4):1927. 10.3390/ijms22041927 33672070 PMC7919680

[68] Williams MS Ziegelstein RC McCann UD Gould NF Ashvetiya T Vaidya D. Platelet serotonin signaling in patients with cardiovascular disease and comorbid depression. Psychosom Med. (2019) 81(4):352–62. 10.1097/psy.0000000000000689 30855555 PMC6499626

[69] Nakatani D Sato H Sakata Y Shiotani I Kinjo K Mizuno H Influence of serotonin transporter gene polymorphism on depressive symptoms and new cardiac events after acute myocardial infarction. Am Heart J. (2005) 150(4):652–8. 10.1016/j.ahj.2005.03.062 16209960

[70] Li Y Cai X Guan Y Wang L Wang S Li Y Adiponectin upregulates mir-133a in cardiac hypertrophy through AMPK activation and reduced ERK1/2 phosphorylation. PLoS One. (2016) 11(2):e0148482. 10.1371/journal.pone.0148482 26845040 PMC4741527

[71] Ding J Jiang C Yang L Wang X. Relationship and effect of mir-1-3p expression and BDNF level in patients with primary hypertension complicated with depression. Cell Mol Biol (Noisy-le-Grand). (2022) 68(1):67–74. 10.14715/cmb/2022.68.1.10 35809326

[72] Cheng C Wang Q You W Chen M Xia J. MiRNAs as biomarkers of myocardial infarction: a meta-analysis. PLoS One. (2014) 9(2):e88566. 10.1371/journal.pone.0088566 24533109 PMC3922900

[73] Liu C Tang M Zhang X Li J Cao G. Knockdown of mir-665 protects against cardiomyocyte ischemia/reperfusion injury-induced ROS accumulation and apoptosis through the activation of Pak1/Akt signaling in myocardial infarction. Int Heart J. (2020) 61(2):347–54. 10.1536/ihj.19-416 32132320

[74] Żurawek D Turecki G. The mirnome of depression. Int J Mol Sci. (2021) 22(21):11312. 10.3390/ijms222111312 34768740 PMC8582693

[75] Bonk S Kirchner K Ameling S Garvert L Völzke H Nauck M APOE ε4 in depression-associated memory impairment-evidence from genetic and MicroRNA analyses. Biomedicines. (2022) 10(7):1560. 10.3390/biomedicines10071560 35884866 PMC9313258

[76] Zheng Z Zeng Y Huang H Xu F. MicroRNA-132 may play a role in coexistence of depression and cardiovascular disease: a hypothesis. Med Sci Monit. (2013) 19:438–43. 10.12659/msm.883935 23748239 PMC3678976

[77] Miguel-Hidalgo JJ Hall KO Bonner H Roller AM Syed M Park CJ MicroRNA-21: expression in oligodendrocytes and correlation with low myelin mRNAs in depression and alcoholism. Prog Neuropsychopharmacol Biol Psychiatry (2017) 79(Pt B):503–14. 10.1016/j.pnpbp.2017.08.009 28802862 PMC5610939

[78] Zhang X Huo Q Sun W Zhang C Wu Z Xing B Rs2910164 in microRNA-146a confers an elevated risk of depression in patients with coronary artery disease by modulating the expression of NOS1. Mol Med Rep. (2018) 18(1):603–9. 10.3892/mmr.2018.8929 29749487

[79] Jiang W Oken H Fiuzat M Shaw LK Martsberger C Kuchibhatla M Plasma omega-3 polyunsaturated fatty acids and survival in patients with chronic heart failure and major depressive disorder. J Cardiovasc Transl Res. (2012) 5(1):92–9. 10.1007/s12265-011-9325-8 22042636

[80] Haberka M Mizia-Stec K Mizia M Gieszczyk K Chmiel A Sitnik-Warchulska K Effects of N-3 polyunsaturated fatty acids on depressive symptoms, anxiety and emotional state in patients with acute myocardial infarction. Pharmacol Rep. (2013) 65(1):59–68. 10.1016/s1734-1140(13)70964-2 23563024

[81] Jain AP Aggarwal KK Zhang PY. Omega-3 fatty acids and cardiovascular disease. Eur Rev Med Pharmacol Sci. (2015) 19(3):441–5. 10.1159/000059783 25720716

[82] Mozaffarian D Wu JH. Omega-3 fatty acids and cardiovascular disease: effects on risk factors, molecular pathways, and clinical events. J Am Coll Cardiol. (2011) 58(20):2047–67. 10.1016/j.jacc.2011.06.063 22051327

[83] Deacon G Kettle C Hayes D Dennis C Tucci J. Omega 3 polyunsaturated fatty acids and the treatment of depression. Crit Rev Food Sci Nutr. (2017) 57(1):212–23. 10.1080/10408398.2013.876959 25830700

[84] Su KP Wang SM Pae CU. Omega-3 polyunsaturated fatty acids for major depressive disorder. Expert Opin Investig Drugs. (2013) 22(12):1519–34. 10.1517/13543784.2013.836487 24083675

[85] Stoll AL Severus WE Freeman MP Rueter S Zboyan HA Diamond E Omega 3 fatty acids in bipolar disorder: a preliminary double-blind, placebo-controlled trial. Arch Gen Psychiatry. (1999) 56(5):407–12. 10.1001/archpsyc.56.5.407 10232294

[86] Harris WS Von Schacky C. The omega-3 index: a new risk factor for death from coronary heart disease? Prev Med. (2004) 39(1):212–20. 10.1016/j.ypmed.2004.02.030 15208005

[87] Parletta N Zarnowiecki D Cho J Wilson A Procter N Gordon A People with schizophrenia and depression have a low omega-3 index. Prostaglandins Leukot Essent Fatty Acids. (2016) 110:42–7. 10.1016/j.plefa.2016.05.007 27255642

[88] Chang JP Chang SS Yang HT Palani M Chen CP Su KP. Polyunsaturated fatty acids (PUFAs) levels in patients with cardiovascular diseases (CVDs) with and without depression. Brain Behav Immun. (2015) 44:28–31. 10.1016/j.bbi.2014.11.005 25452150

[89] Liu H Zhuang J Tang P Li J Xiong X Deng H. The role of the gut microbiota in coronary heart disease. Curr Atheroscler Rep. (2020) 22(12):77. 10.1007/s11883-020-00892-2 33063240

[90] Alam MJ Puppala V Uppulapu SK Das B Banerjee SK. Human microbiome and cardiovascular diseases. Prog Mol Biol Transl Sci. (2022) 192(1):231–79. 10.1016/bs.pmbts.2022.07.012 36280321

[91] Witkowski M Weeks TL Hazen SL. Gut microbiota and cardiovascular disease. Circ Res. (2020) 127(4):553–70. 10.1161/circresaha.120.316242 32762536 PMC7416843

[92] Jie Z Xia H Zhong SL Feng Q Li S Liang S The gut microbiome in atherosclerotic cardiovascular disease. Nat Commun. (2017) 8(1):845. 10.1038/s41467-017-00900-1 29018189 PMC5635030

[93] Zhu F Tu H Chen T. The microbiota-gut-brain axis in depression: the potential pathophysiological mechanisms and microbiota combined antidepression effect. Nutrients. (2022) 14(10):2081. 10.3390/nu14102081 35631224 PMC9144102

[94] Tyagi P Tasleem M Prakash S Chouhan G. Intermingling of gut microbiota with brain: exploring the role of probiotics in battle against depressive disorders. Food Res Int. (2020) 137:109489. 10.1016/j.foodres.2020.109489 33233143

[95] Simpson CA Diaz-Arteche C Eliby D Schwartz OS Simmons JG Cowan CSM. The gut microbiota in anxiety and depression—a systematic review. Clin Psychol Rev. (2021) 83:101943. 10.1016/j.cpr.2020.101943 33271426

[96] Li D Liu R Wang M Peng R Fu S Fu A 3β-hydroxysteroid dehydrogenase expressed by gut microbes degrades testosterone and is linked to depression in males. Cell Host Microbe. (2022) 30(3):329–39.e5. 10.1016/j.chom.2022.01.001 35108497

[97] Amare AT Schubert KO Klingler-Hoffmann M Cohen-Woods S Baune BT. The genetic overlap between mood disorders and cardiometabolic diseases: a systematic review of genome wide and candidate gene studies. Transl Psychiatry. (2017) 7(1):e1007. 10.1038/tp.2016.261 28117839 PMC5545727

[98] McCaffery JM Frasure-Smith N Dubé MP Théroux P Rouleau GA Duan Q Common genetic vulnerability to depressive symptoms and coronary artery disease: a review and development of candidate genes related to inflammation and serotonin. Psychosom Med. (2006) 68(2):187–200. 10.1097/01.psy.0000208630.79271.a0 16554382

[99] Lu Y Wang Z Georgakis MK Lin H Zheng L. Genetic liability to depression and risk of coronary artery disease, myocardial infarction, and other cardiovascular outcomes. J Am Heart Assoc. (2021) 10(1):e017986. 10.1161/jaha.120.017986 33372528 PMC7955472

[100] Huang K Zhang X Duan J Wang R Wu Z Yang C STAT4 and COL1A2 are potential diagnostic biomarkers and therapeutic targets for heart failure comorbided with depression. Brain Res Bull. (2022) 184:68–75. 10.1016/j.brainresbull.2022.03.014 35367598

[101] Torgersen K Rahman Z Bahrami S Hindley GFL Parker N Frei O Shared genetic loci between depression and cardiometabolic traits. PLoS Genet. (2022) 18(5):e1010161. 10.1371/journal.pgen.1010161 35560157 PMC9170110

[102] Redlich C Berk M Williams LJ Sundquist J Sundquist K Li X. Statin use and risk of depression: a Swedish national cohort study. BMC Psychiatry. (2014) 14:348. 10.1186/s12888-014-0348-y 25471121 PMC4266881

[103] Baldwin DS Necking O Schmidt SN Ren H Reines EH. Efficacy and safety of vortioxetine in treatment of patients with major depressive disorder and common co-morbid physical illness. J Affect Disord. (2022) 311:588–94. 10.1016/j.jad.2022.05.098 35597471

[104] Hansen BH Hanash JA Rasmussen A Hansen JF Andersen NL Nielsen OW Effects of escitalopram in prevention of depression in patients with acute coronary syndrome (DECARD). J Psychosom Res. (2012) 72(1):11–6. 10.1016/j.jpsychores.2011.07.001 22200516

[105] Honig A Kuyper AM Schene AH van Melle JP de Jonge P Tulner DM Treatment of post-myocardial infarction depressive disorder: a randomized, placebo-controlled trial with mirtazapine. Psychosom Med. (2007) 69(7):606–13. 10.1097/PSY.0b013e31814b260d 17846258

[106] Sun Y Wang Z Wang C Tang Z Zhao H. Psycho-cardiology therapeutic effects of Shuangxinfang in rats with depression-behavior post acute myocardial infarction: focus on protein S100A9 from proteomics. Biomed Pharmacother. (2021) 144:112303. 10.1016/j.biopha.2021.112303 34673424

[107] Hou J Wang C Ma D Chen Y Jin H An Y The cardioprotective and anxiolytic effects of Chaihujialonggumuli granule on rats with anxiety after acute myocardial infarction is partly mediated by suppression of CXCR4/NF-κB/GSDMD pathway. Biomed Pharmacother. (2021) 133:111015. 10.1016/j.biopha.2020.111015 33232924

[108] Jiang B Wu RM Li HD Li K Li H Dang WZ Yixin Ningshen tablet alleviates comorbidity of myocardial infarction and depression by enhancing myocardial energy metabolism and increasing availability of monoamine neurotransmitter. Chin J Integr Med. (2022) 28(7):586–93. 10.1007/s11655-022-3570-3 35319073

[109] Turner KM Winder R Campbell JL Richards DA Gandhi M Dickens CM Patients’ and nurses’ views on providing psychological support within cardiac rehabilitation programmes: a qualitative study. BMJ Open. (2017) 7(9):e017510. 10.1136/bmjopen-2017-017510 28864707 PMC5589022

[110] Westas M Lundgren J Andersson G Mourad G Johansson P. Effects of internet-delivered cognitive behavioural therapy adapted for patients with cardiovascular disease and depression: a long-term follow-up of a randomized controlled trial at 6 and 12 months posttreatment. Eur J Cardiovasc Nurs. (2022) 21(6):559–67. 10.1093/eurjcn/zvab131 35061868

[111] Huffman JC Mastromauro CA Sowden G Fricchione GL Healy BC Januzzi JL. Impact of a depression care management program for hospitalized cardiac patients. Circ Cardiovasc Qual Outcomes. (2011) 4(2):198–205. 10.1161/circoutcomes.110.959379 21386067

[112] Zheng X Zheng Y Ma J Zhang M Zhang Y Liu X Effect of exercise-based cardiac rehabilitation on anxiety and depression in patients with myocardial infarction: a systematic review and meta-analysis. Heart Lung. (2019) 48(1):1–7. 10.1016/j.hrtlng.2018.09.011 30366575

